# 

**Published:** 1882-06-20

**Authors:** 


### TABLE OF CONTENTS.

ORIGINAL AND SELECTED ARTICLES.

Remarks on Hotz's Operation for Entropion and Trichiasis, with seven cases, by A. G. Hobbs, M. D, of Atlanta, 201. Syphilitic Ulceration of the Eye-Lid (conjunctiva) in the Infant, by A. W. Calhoun, M. D., of Atlanta, 207. Cases in Practice, by A. A. Davidson, M. D., of Milan, Tenn, 209. Grape Sugar and Glucose, by J. M. Tallman, 210. Remarks on Intestinal Parasites, by F. P. Henry, M. D., 213. The Treatment of Diphtheria in the past eight years, 216. The Duties of Practitioners in Relation to their Professional Services to Each Other, to their Families, Widows and Children, 218.

ABSTRACTS AND GLEANINGS.

The Parasitic Nature of Tubercular Consumption, 219. Prolonged Gestation, 220. Vehicle for Salycilic Acid—Treatment of Hay Fever, 221. Effect of an Overdose of Podophyllin—Therapeutic Effects of Damiana, 222. Vomiting in Pregnancy, 223. Bandage After Delivery—Typhoid Diarrhœa, 224. Treatment of Acute Dysentery with Aconite—Leibig's Corn Cure, 225. Rules of Practice in Operating upon Pregnant Women—Cholera Infantum—Antidote for Strychnine, 226. Treatment of Rabies with Hoang-Nan—The Proper Dose of Conium—Relief of Pain in Lead Colic, 227. Can a Man have Syphilis Twice—Wight on the Combatting of Small-Pox, 228. Micro-Organisms and Disease—Pruritus Ani—Jamiaca Dogwood—Treatment of Gonorrhœa, 229. Incompatibility of Salicylate of Sodium with Spirits of Nitrous Æther—Beef Tea and Coca—Giteau's Body—Cause of the Decay of Teeth—Chloral Enemata in Vomiting of Pregnancy—Experts' Fees in Courts of Justice, 230.

SCIENTIFIC ITEMS.

The Telephone—How Fast Does Niagara Recede—Unexplored Countries—Effect of Compression on Solids, 231. M. Pasteur, 232.

PRACTICAL NOTES AND FORMULÆ.

Cinchonic Mixtures; Treatment of Eczema; Diarrhœa, 233. Treatment of Uterine Fibroids; Hope's Camphor Mixture; Pills for Constipation; Treatment of Dysentery; Blue Ointment for Ring-Worm; Treatment of Laryngeal Phthisis, 234. Oil of Turpentine in Mixtures; Hardy's Ointment; Pills for Dyspepsia; Arsenical Treatment of Chorea, 235. New Treatment for Vaginitis; Boils; Quinidia Combination for Intermittents; Hot Water for the Heart; Quinia in the Treatment of Cholera Infantum, 236.

EDITORIALS AND MISCELLANEOUS.

Editorial Notices, 237. Plank Side Walks; Southern Medical College Hospital; Fourth Annual Report of the Board of Health, of Augusta, Ga., 238. American Medical Association; Journalism in the South, 239. Receipted; Special Notices, 240.

